# Do Automated Peritoneal Dialysis and Continuous Ambulatory Peritoneal Dialysis Have the Same Clinical Outcomes? A Ten-year Cohort Study in Taiwan

**DOI:** 10.1038/srep29276

**Published:** 2016-07-08

**Authors:** Chao-Hsiun Tang, Tso-Hsiao Chen, Te-Chao Fang, Siao-Yuan Huang, Kuan-Chih Huang, Yu-Ting Wu, Chia-Chen Wang, Yuh-Mou Sue

**Affiliations:** 1 School of Health Care Administration, College of Management, Taipei Medical University, Taipei, Taiwan; 2 Division of Nephrology, Department of Internal Medicine, School of Medicine, College of Medicine, Taipei Medical University, Taipei, Taiwan; 3 Division of Nephrology, Department of Internal Medicine, Wan Fang Hospital, Taipei Medical University, Taipei, Taiwan; 4 School of Medicine, Fu-Jen Catholic University, New Taipei City, Taiwan

## Abstract

This paper reports a comprehensive comparison for mortality and technique failure rates between automated peritoneal dialysis (APD) and continuous ambulatory peritoneal dialysis (CAPD) in Taiwan. A propensity-score matched cohort study was conducted by retrieving APD and CAPD patients identified from the Taiwan National Health Insurance Research Database between 2001 and 2010. The main outcomes were the 5-year mortality and technique failure rates. Further analyses were then carried out based upon the first (2001–2004), second (2005–2007), and third (2008–2010) sub-periods. Similar baseline characteristics were identified for APD (n = 2,287) and CAPD (n = 2,287) patients. The proportion on APD therapy increased rapidly in the second sub-period. As compared to CAPD patients of this sub-period, APD patients had a significantly higher risk of mortality (HR, 1.37; 95% CI 1.09–1.72; *p* < 0.01) and technique failure (HR, 1.43; 95% CI, 1.10–1.86; *p* < 0.01), particularly in the first year after peritoneal dialysis commencement. However, APD patients had similar mortality and technique failure rates to those of CAPD patients throughout the full sample period and the first and third sub-periods. These findings do not suggest the presence of a clear advantage of CAPD over APD. Differences observed between these two modalities might be attributed to specials circumstances of sub-periods.

Mortality is one of the most important outcomes to be taken into consideration when selecting dialysis modalities amongst patients entering ‘end-stage renal disease’ (ESRD). Recent observational studies suggest that the survival of peritoneal dialysis (PD) patients has improved over time, and is now comparable to the survival of hemodialysis (HD) patients^1^,^2^,^3^. However, given that the incidence and prevalence of ESRD in Taiwan are amongst the highest in the world, the increase has become an increasing financial burden on the Taiwan National Health Insurance (NHI) system^4^,^5^; and in deed, by 2014, the costs of dialysis were accounting for an astonishing 7.4% of the total annual NHI expenditure in Taiwan^6^. Since PD has a similar all-cause mortality (ACM) rate to that of HD, but with lower medical costs, the Taiwan NHI Administration has been promoting the utilization of PD as a viable alternative since 2005^3^,^7^,^8^; and indeed, over the years, the Taiwan NHI administration has been gradually introducing a progressive program of reductions in the reimbursement rates for HD and a corresponding program of increases in the reimbursement rates for PD.

In order to promote the more widespread use of PD, from May 2008 onwards, the Taiwan NHI payment scheme not only covered continuous ambulatory PD (CAPD) but also was extended to cover the machine costs of automated PD (APD); these are the two most frequently used PD modalities. As a result, there has been a gradually increasing trend in the prevalence of PD usage in Taiwan (including APD and CAPD) from 6.5% in 2003, to 8.5% in 2007 and 10.3% in 2009^5^,^9^. At the same time, the number of patients treated with PD on a global scale has also risen considerably, with a 2.5-fold increase in the prevalence of PD patients in developing countries between 1997 and 2008^10^.

There is, however, a distinct lack of evidence on the mortality rates of both APD and CAPD modalities in eastern countries, which is clearly of importance in enabling healthcare providers to select the appropriate PD modality. Previous ESRD registries have documented racial differences in the crude mortality rates of patients on dialysis^11^,^12^, and whist the clinical outcomes of the two PD modalities have been compared in western countries, the ACM results have been somewhat inconsistent^13^,^14^,^15^,^16^,^17^,^18^. Three multicenter studies in western countries comparing ACM and technique failure (TF) rates between APD and CAPD methods reported similar outcomes^13^,^14^,^15^. A further study carried out in the US found that APD patients had a better technique survival rate, but no significant difference in ACM was identified between APD and CAPD patients^16^. Two studies undertaken in Mexico and Brazil also reported that as compared to CAPD patients, APD patients had a better technique survival rate and significantly lower ACM^17^,^19^.

Several factors may have potentially contributed to the variations in the results reported in the western studies, including geographic variations, racial and ethnic differences, patient choice bias, physician bias and differences in insurance payment scheme for APD modality; and indeed, APD has the distinct characteristic of short dwell times under automated devices, with such usage having increased over recent years. Given the increasing use of both APD and CAPD in Taiwan, an examination of the comparative assessment of the survival rates of the two PD modalities has now become a crucial issue. Our population-based study has therefore been designed to facilitate a comprehensive comparison for ACM and TF rates amongst these two PD modalities.

## Results

### Demographic characteristics

**Figure 1:**
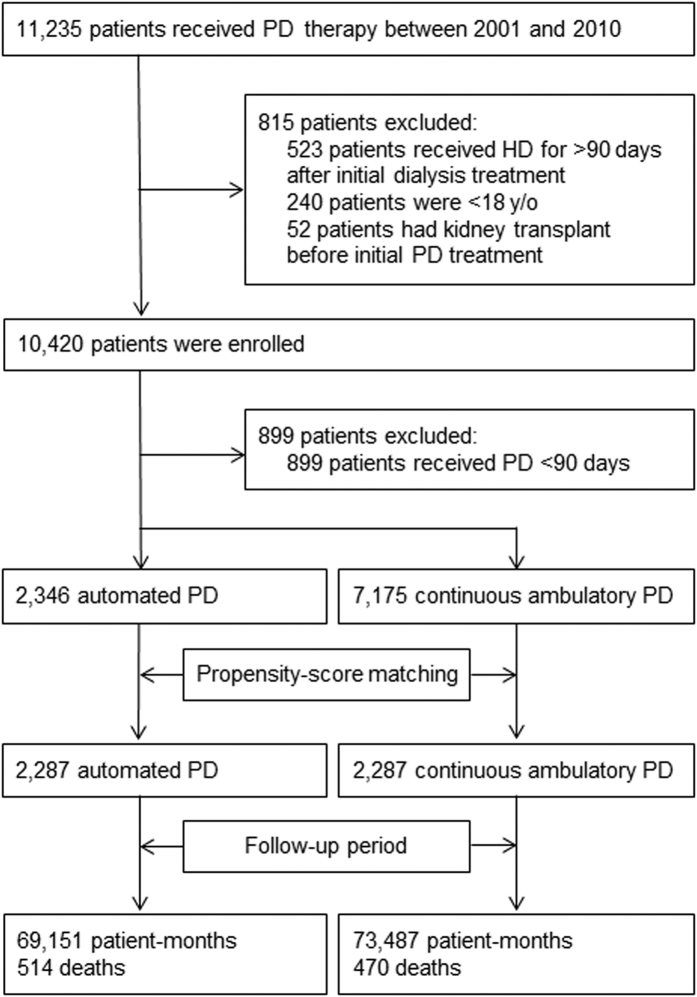
Enrollment of study participants.

**Figure 2:**
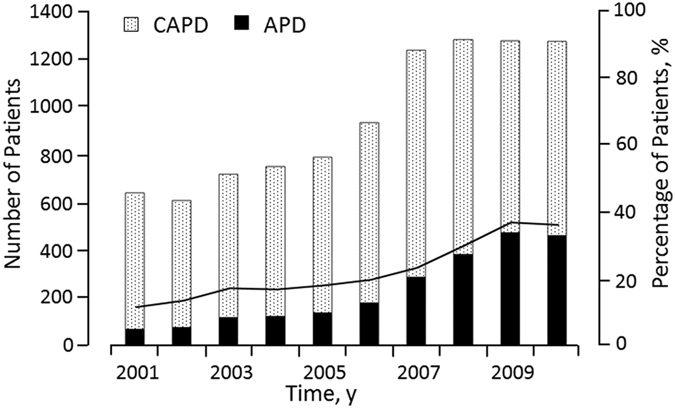
Total numbers of APD and CAPD patients (bar chart) and percentage of APD patients to total PD patients (solid line).

As shown in Fig. 1, our APD patient cohort (n = 2,346) and CAPD patient cohort (n = 7,175) were originally enrolled into the full sample between 2001 and 2010; however, as a result of the 1:1 propensity-score matching analyses, 2,287 APD and 2,287 CAPD patients were selected for subsequent analysis. The baseline patient characteristics are reported in Table 1. Following the propensity-score matching, each group, based upon age and year at cohort entry, contained the same number of APD and CAPD patients. The other baseline characteristics were found to be similar for both groups with the one exception of cirrhosis of the liver (Table 1). Further analyses were then carried out based upon the first (2001–2004), second (2005–2007), and third (2008–2010) sub-periods. Figure 2 shows the overall cohort number of PD patients. As compared to the first and third sub-periods, a much more rapid increase is discernible in the number of PD patients in the second sub-period (791 patients in 2005, reaching a peak of 1,281 patients in 2008). This sub-period coincides with the promotion of the use of PD by the Taiwan NHI Administration. Figure 2 also shows the percentage of APD patients to the overall cohort of PD patients by year at cohort entry, with a discernible gradual increase in the proportion of APD patients from 11.4% in 2001 to 18% in 2005, reaching a peak of 37.2% in 2009.

### Univariate analyses for all-cause mortality and technique failure

The primary outcomes after matching (as reported in Table 2) show that during the 2001–2010 sample period, a total of 514 (22.5%) APD patients had died, which was slightly higher than the 470 (20.6%) deaths that had occurred amongst the CAPD patients. It is also noted from Table 2 that greater numbers of APD patients had received kidney transplants than CAPD patients during the 2001–2010 period and the first sub-period. The incident rate of ACM was higher amongst APD patients than CAPD patients in the second sub-period, with the comparable incident rate of TF also revealed a consistent result. The events of peritonitis per 1,000 patient-years were significantly lower amongst APD patients than CAPD patients during the first and third sub-periods, with the exception of the second sub-period (RR, 1.08; 95% CI, 0.93–1.24). Increase home visits of APD patients were noted from the second (22.5%) to third (24.6%) sub-period. CAPD patients also had the same trend (25.5% to 28.8%). Supplementary Table S3 shows annual peritoneal dialysis-related quantity of solution of APD and CAPD patients. The total quantities of 1.5% and 2.5% dextrose solution were significantly higher amongst APD patients than CAPD patients.

**Figure 3:**
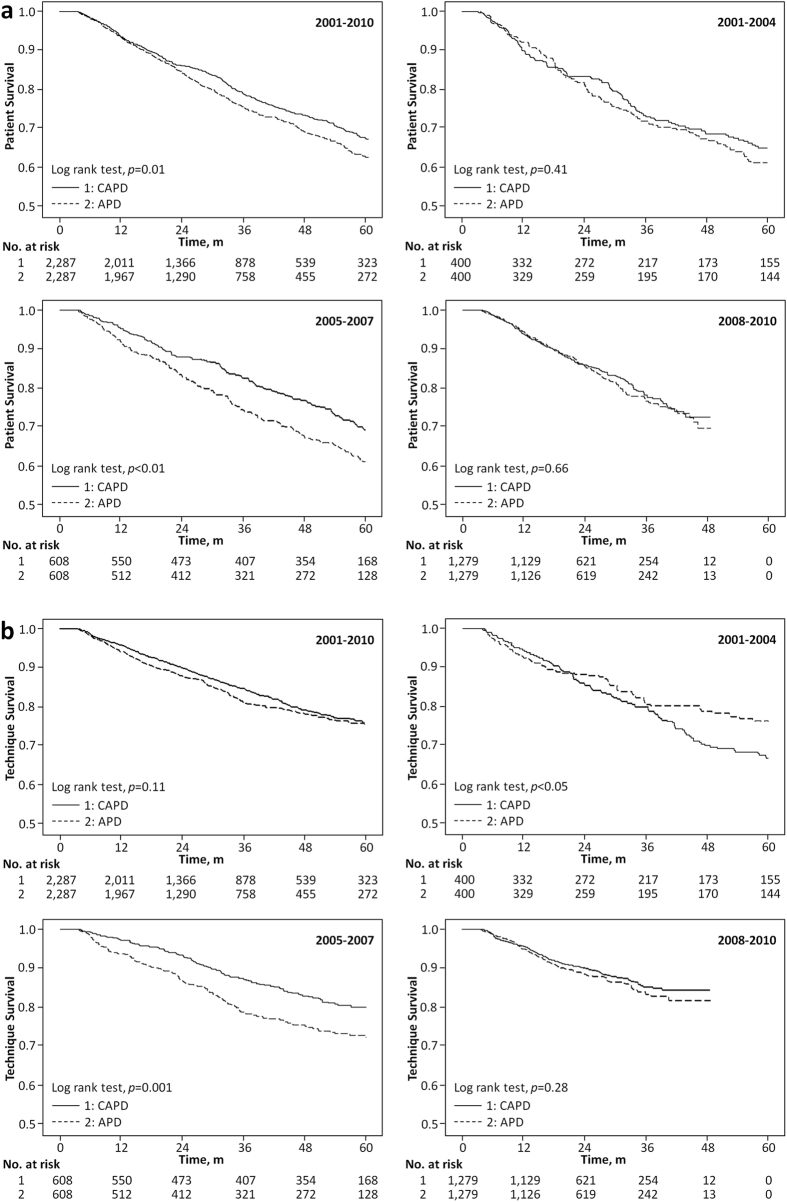
Kaplan-Meier analyses of (**a**) patient survival and (**b**) technique survival probabilities, by cohort periods.

The Kaplan-Meier curves for the patient survival rates (technique survival rates) during the overall sample period and the three sub-periods are illustrated in Fig. 3a,b), from which we can see that the survival benefit for APD patients is inferior to that of the CAPD patients during the overall sample period (p = 0.01) and the second sub-period (p < 0.01). Similar technique survival rates are discernible for both the APD and CAPD patients during the overall sample period; however, separate comparisons of each of the sub-period reveal that APD patients had an inferior technique survival rate in the second sub-period (p = 0.001).

### Multivariable analyses for all-cause mortality and technique failure

We subsequently carried out Cox proportional hazard regressions and competing risks analyses to facilitate our examination of the differences on the ACM and TF rates between the APD and CAPD patients (Table 3). As compared to the CAPD patients, the APD patients were found to have a slightly higher risk of ACM and TF with HRs of 1.17 (95% CI, 1.03–1.32; *p* = 0.02) and 1.17 (95% CI, 1.01–1.36; *p* = 0.04) after Cox proportional hazard regressions. However, the results of competing risks analyses showed the APD and CAPD patients had similar risks of ACM (HR, 1.12; 95% CI: 0.98–1.27; *p* = 0.09) and TF (HR, 1.11; 95% CI: 0.96–1.29; *p* = 0.16) after multivariate adjustment. A further comparison was subsequently undertaken between the ACM and TF rates for APD vis-à-vis CAPD patients in the three sub-periods. The results on ACM, after multivariate adjustment, revealed that the APD patients had a significantly higher risk of ACM than the CAPD patients in only the second sub-period (HR, 1.51; 95% CI: 1.22–1.88; *p* < 0.001). As regards the results on TF, as compared to the CAPD patients, the APD patients were found to have a significantly lower TF rate in the first sub-period (HR, 0.70; 95% CI: 0.52-0.95; p = 0.02) but a significantly higher TF rate in the second sub-period (HR, 1.51; 95% CI: 1.22–1.88; *p* < 0.001). In addition, the results of the competing risks analyses were similar with the results of Cox proportional hazard regressions in the three sub-periods (Table 3). We also carried out sub-group Cox proportional hazard regression analyses for the ACM and TF rates of the APD and CAPD patients using the first sub-period as the reference (Supplement Table S4). The results revealed that as compared to the first sub-period, CAPD patients had a lower risk of ACM and TF in the other two sub-periods; however, no similar trend was discernible for the APD patients.

### APD-to-CAPD hazard ratios by sample period and age

**Figure 4:**
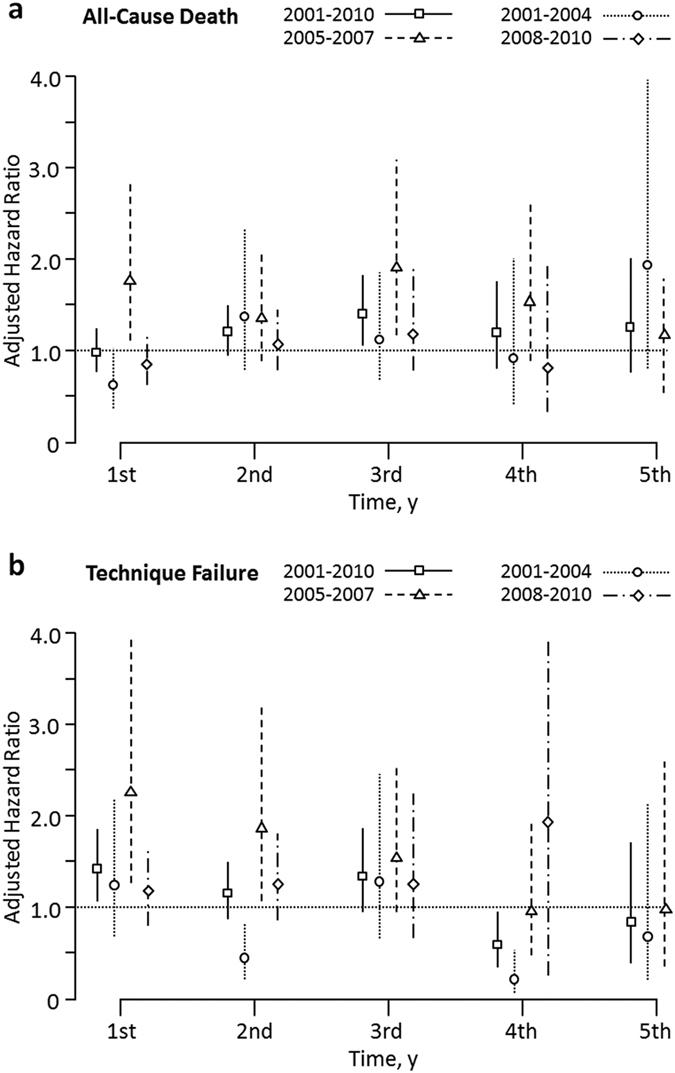
Adjusted hazard ratios of APD to CAPD from the final multivariate model for (**a**) all-cause mortality and (**b**) technique failure, by year. The APD patients had a significantly higher risk of all-cause mortality in the 2005–2007 sub-period, particularly in the 1^st^ and 3^rd^ years. In contrast, a lower risk of all-cause mortality in the 2001–2004 sub-period was found, particularly in the 1^st^ year. The adjusted hazard ratios of the technique failure fluctuated with a notably higher risk in the 1^st^ and 2^nd^ years of the 2005–2007 sub-period and a considerably lower risk in the 2^nd^ and 4^th^ years of the 2001–2004 sub-period. Patients were followed till December 31, 2011.

Figure 4a,b illustrate the adjusted HRs of APD to CAPD after adjusted patient demographics, comorbidities, events of peritonitis and icodextrin usage for all the patients. APD patients had a significantly higher risk of both ACM and TF in the second sub-period, particularly in the first year after the commencement of PD therapy. The adjusted HRs of the TF in the first sub-period fluctuated with inconsistent risk during the follow-up period.

The HRs of APD to CAPD patients with regard to ACM and TF after multivariate adjustment by age groups are illustrated in the supplementary Fig. S1. Compared to CAPD patients, APD patients had a similar risk of ACM and TF in all age groups except aged 60 to 69 years, with APD patients having a high risk of ACM (HR, 1.68; 95% CI, 1.28–2.20; *p* < 0.001) and TF (HR, 1.58; 95% CI, 1.15–2.16; *p* = 0.01).

### Sensitivity analyses for all-cause mortality and technique failure

As shown in Table 4, the results of the sub-group analyses on pure APD patients and pure CAPD patients were found to be similar to the primary analyses in the full sample period and the three sub-periods. The results after Cox proportional hazard regression revealed that APD patients still had a significantly higher risk of ACM and TF than CAPD patients in the full sample period and second sub-period. Similar results were obtained for ACM and TF risk using an alternative definition of delaying the index date to 120 and 180 days and regrouping the APD or CAPD patients. The results are shown in the supplementary tables (Tables S5 and S6). Furthermore, the results of competing risks analyses revealed similar risks of ACM and TF with the results of Cox proportional hazard regression except the result of TF in the full sample period, which showed no notable difference between APD and CAPD patients (HR, 1.15; 95% CI 0.98–1.33; *p* = 0.08).

## Discussion

The results of our national study of a propensity-score matched cohort of patients in receipt of APD and CAPD between 2001 and 2010 reveal that both the APD and CAPD patients had similar ACM and TF rates throughout the full sample period and the first and third sub-periods; however, APD patients were found to have a significantly higher risk of both ACM and TF in the second sub-period, particularly in the first year after the commencement of PD therapy. These results, based upon NHIRD data, remain robust under various types of sensitivity analyses. We have also found that as compared to CAPD patients, APD patients had a lower risk of developing peritonitis with the exception of the second sub-period.

There has been a considerable increase in the overall prevalence of PD patients in Taiwan ever since 2005, which was the year in which the Taiwan NHI Administration started promoting greater PD usage; furthermore, from 2008 onwards, reimbursements received from the Taiwan NHI payment scheme were also extended to cover the machine costs of APD. The impact of the policy is not only reflected in the increase in the total number of PD patients but also in the remarkable growth in the number of patients choosing APD as their preferred PD therapy, from about 18% of incident PD patients in 2005 to 30.1% in 2008, and peaking at 37.2% in 2009 (Fig. 2). This trend has also been reported on a global scale; for example, from 1997 to 2008, the use of APD in several developing countries is reported to have increased by 14.5%, whilst in the more developed countries APD usage was up by 30.3%^10^. In the United States and Canada, the proportion of PD patients treated under the APD modality has been increasingly rapidly, to more than 60%, essentially because the APD modality provides better quality of life and frees the patients for most of their waking hours^20^,^21^,^22^. In addition to coverage of APD machine costs under the NHI payment scheme, other reasons for the selection of the APD modality as the preferred PD therapy include the convenience of the APD system and better mental health quality under APD usage^10^,^22^,^23^.

The evidence on the mortality rates associated with APD and CAPD modalities in East Asia countries is distinctly lacking. Our findings based on the Taiwan NHIRD reveal a significantly higher risk of ACM and TF for APD patients in the second sub-period, particularly in the first year after the commencement of PD therapy (Fig. 3a). The results differ from the findings of other multi-center studies which found no significant differences in ACM and TF rates between the two modalities^13^,^14^,^15^. As shown in supplementary Table S4, CAPD patients had significantly better survival rates in the second and third sub-periods than in the first sub-period, although this trend was not discernible for APD patients in the second sub-period. The impact of the NHI payment scheme on PD therapy led to rapid growth in the number of patients during the second sub-period, raising concerns with regard to the lack of sufficient nursing experience to care for the growing patient numbers. Potential physician choice bias under the new payment scheme by allocating patients towards a particular dialysis modality may also occur. Under the new payment scheme, patients with insufficient self-care ability may have been encouraged to select APD therapy through such bias essentially because the peritonitis rate ratio between APD and CAPD patients was found to be at its highest during this period (Table 2).

The choice to implement CAPD or APD should not only take into account the rate of peritoneal solute transport but also patient preference and clinical condition. According to the European Best Practice Guidelines published in 2005, APD is indicated in (i) inability to achieve efficient blood purification and/or ultrafiltration by means of CAPD; (ii) need to prevent high intraperitoneal pressure; and (iii) patient preference, whereas the prescription of CAPD is well established in patients with a low rate of peritoneal solute transport^24^. Unfortunately, some important laboratory data regarding the choice of modality and mortality, such as peritoneal transport status, residual renal function, serum albumin level, weekly KT/V and weekly creatinine clearance were not available in this study because the NHIRD does not contain laboratory results. We suggested that the increased number of APD patients during the second sub-period could well be due to high transporters essentially because APD modality is more suitable for these patients^25^. A meta-analysis study has shown that there was a higher risk of death in high transporters treated with APD as compared to those treated with CAPD (relative risk, 1.15; 95% CI: 1.07–1.23; p < 0.001)^26^. As such, in the case of limitations of the NHIRD, some potential impact factors could not be adjusted and these patients may well have been over-represented in the APD patient cohort.

The major difference between APD and CAPD procedures include the methods and frequency of setting up manual connections and disconnections from the PD catheter and the dialysate bags, with APD having fewer dialysate exchanges than CAPD; however, the results of our study show that there was a higher risk of TF in the APD modality during the second sub-period, a result which is not consistent with the other two sub-periods or the results of prior multi-center studies in different parts of the world^13^,^14^,^15^. Ever since the Y-set (twin-bag) connecting system was introduced into Taiwan in 1997, it has become standard practice for CAPD therapy, which means that its usage is encompassed in the full period examined in the present study. It has also been the main reason for the prevention of peritonitis in CAPD^27^. A prior single-center study in Taiwan comparing APD with the twin-bag system revealed lower incidences of peritonitis with the use of the APD (146.4 events/1,000 patient-years vs. 273.6 events/1,000 patient-years, p < 0.001)^28^. Our national cohort study also reveals that as compared to CAPD patients, APD patients had a lower peritonitis incidence rate ratio in all of the sample periods, with the exception of the second sub-period (Table 2), which could be the major cause of the higher TF rate found in APD patients during the same period. Our sub-group analysis also revealed that APD patients had a higher risk of TF rate in the first two years (Fig. 4b), which indicates that the rapid increase in the numbers of PD patients choosing the APD modality in the second sub-period also resulted in them returning to the HD modality in the first two years. The higher risk of TF rate in APD patients returned to a similar risk level in the third sub-period once the rapid growth in PD patients had subsided. Given the increasing use of APD in Taiwan, it is particularly important to clarify the outcome differences between APD and CAPD patients. We therefore suggest that the significant differences in the risk of ACM and TF between APD and CAPD patients in the second sub-period could be attributable to the impact of the Taiwan NHI payment scheme and the rapid growth in the total numbers of PD patients. The trend of rapid increase in the number of incident PD patient flattened since 2008 probably owing to the detected higher TF rate during the second sub-period by physicians, less promotion of PD therapy by the Taiwan NHI Administration, and the intervention of increased home visits by better experienced nurses.

The main strengths of this study include the use of a national population-based sample and propensity-score matched cohort study involving an East Asian population. However, despite these strengths, these data sources also have some limitations, which largely coincide with those commonly found in administrative database research studies. First, the underlying diseases causing ESRD were unknown and the assignment of patients to the two PD therapies was not random; thus, caution is urged in attempting to infer causality. Second, detailed laboratory results were not included in the NHIRD. So we could not use the assigned ICD-9-CM codes to identify the albumin level, residual renal function, nutritional status, ultrafiltration rate, peritoneal function test and dialysis clearance (weekly KT/V and weekly creatinine clearance), which might be associated with mortality. Third, the CAPD patients selected for this study were those who matched the APD patients; however, some characteristics of the selected CAPD patients may differ from the general population of CAPD patients. Other residual confounding may have been present, even after adjustment for the most relevant covariables; for example, we could not identify from the Taiwan NHIRD how patients or physicians chose the APD or CAPD modality at the time of treatment-modality decision, the self-care ability of PD patients after starting PD, the support system within their family, the educational process of PD or the therapeutic compliance of the PD patients. These biases were inherent to PD modality prescription and could not be overcome without a randomized controlled trial.

In conclusion, this study clarifies the overall survival benefits of APD and CAPD in an East Asian population based upon the use of the Taiwan NHIRD. The APD patients had similar ACM and TF rates as CAPD patients, except in the second sub-period, particularly in the first year after the commencement of PD therapy, which is clearly associated with the rapid growth in the overall number of PD patients. The findings of the study do not suggest the presence of a clear advantage of CAPD over APD. Differences observed between modalities might be attributed to specials circumstances of sample periods. Prospective randomized studies are needed to verify these findings.

## Methods

### Study design and data sources

We conducted a nationwide retrospective cohort study by retrieving data from the Taiwan NHI Research Database (NHIRD) on all patients in receipt of PD between January 1, 1999 and December 31, 2011. The NHIRD contains healthcare data on over 99% of the entire population of Taiwan (23 million in population); indeed, this database is one of the largest and most comprehensive databases in the world, encompassing information on virtually all patients in receipt of renal replacement therapy in Taiwan between 1995 and 2011. The dataset adopted for this study has been widely utilized for epidemiologic research with the results having been validated for both chronic kidney disease and ESRD^29^. Comprehensive details on the files used from the NHIRD have already been provided in our previous work^30^. Each of the diseases examined in this study was defined using the International Classification of Diseases, 9th Revision, Clinical Modification (ICD-9-CM) codes and the corresponding types of treatment received. The Joint Institutional Review Board of Taipei Medical University approved this study, and the informed consent was waived as a result of the personal information obtained from the NHIRD having been de-identified.

### Study cohort and participants

All ESRD patients with long-term PD were identified from the registry for catastrophic illnesses; those ESRD patients in need of long-term dialysis in Taiwan, who are required to be confirmed by two nephrologists as suffering from a catastrophic illness according to the NHI Administration, are subsequently exempted from co-payments under the NHI system. Long-term PD patients were defined as those who had received PD therapy as their dialysis modality 90 days after the first dialysis commencement and continued for at least 90 days between January 1, 2001 and December 31, 2010. Baseline characteristics were collected on the sample patients, with these patients subsequently being followed up by referring to the NHIRD database covering the period from January 1, 1999 to December 31, 2011. Patients were excluded from the sample if: (1) they were less than 18 years of age; (2) they had received HD more than 90 days after their initial dialysis treatment; or (3) they were in receipt of a kidney transplant prior to commencing PD treatment. The patients were then categorized as APD or CAPD patients according to whether or not they were in receipt of PD therapy via a cycler for at least 90 days after PD commencement. The types of APD patients enrolled into the present study included: (1) nocturnal intermittent PD with an empty cavity during the day; (2) continuous optimized PD with one or two exchanges before the night session; (3) continuous cycling PD with a full peritoneal cavity during the day; and (4) tidal PD. The index date was defined as the first day on which the corresponding patient first received either APD or CAPD therapy for a period lasting at least 90 days. A total of 2,346 APD and 7,175 CAPD therapy patients were included in this study. Figure 1 provides a schematic diagram of the study sample along with the exclusion criteria.

### Propensity score computation and matching

The computation of the propensity scores was based upon the variables listed in Table 1, including patient age and gender, number of hospitalizations before enrollment, Charlson comorbidity index score, comorbidities, medications received at study enrollment, and premium wage classes. The comorbidities were defined as at least three outpatient visit claims or one claim for incident hospitalization. All baseline covariates were extracted from the assigned period during which the subjects were identified (within 3 months to 2 years prior to the index date) (Table 1).

The logistic regression model used in this study for both the calculation and distribution of the propensity scores amongst APD and CAPD patients are described in the supplementary tables (Tables S1 and S2). Although the full sample size ratio of APD to CAPD differed before matching (about 1:3), the ratio increased gradually every year (from near 1:8 in 2001 to less than 1:2 in 2010) (Fig. 2). In order to match by both age group and year at cohort entry, the APD and CAPD patients were 1:1 matched according to the ‘nearest available matching without replacement’ on the estimated propensity score and based upon a ≤0.1 difference in their propensity scores (Fig. 1 and Table 1)^31^.

### Outcome measures

The main outcomes of interest to this study are the ACM rate and the TF rate during the five-year period after the index date. For all of the sample patients under APD or CAPD therapy, the censoring criteria established for this study were that the patients were followed-up for up to 5 years until the last day of follow-up (December 31, 2011), transferred to maintenance HD for at least 60 days, in receipt of a kidney transplant or death (whichever happened first). Given that switching between APD and CAPD modalities during the follow-up period is attributed to the original PD therapy, it was not subjected to censoring.

Since the NHI in Taiwan is a compulsory program, those patients on whom follow-up had failed had invariably died, or in very rare cases, moved abroad. Cases of the death in this study were therefore identified as: (1) the date of death was available and obtained from the NHIRD; or (2) the patients had been withdrawn from the NHI program and had not been enrolled in the NHI beneficiary registry files. If the death of a patient occurred within 60 days after switching from PD to maintenance HD, then the death was attributed to the original PD modality; conversely, if the death of a patient occurred within 60 days of a kidney transplant, then the death was attributed to the transplant.

TF was defined in the present study as a transfer from PD to maintenance HD for at least 60 days, with the examination being undertaken without taking into account any occurrences of death. We also calculated the cumulated episodes of PD-related peritonitis for each patient during the follow-up periods, with peritonitis being defined using ICD-9-CM codes 567.9 and 996.59 and concurrent antibiotics treatment.

### Statistical analysis

Patients undergoing APD and CAPD were compared at the baseline, with the patient cohorts being divided into three sub-periods, 2001–2004, 2005–2007, and 2008–2010 based upon the index date, in order to observe any changes over time. We used the independent sample Student *t*-test to examine normally-distributed continuous variables and the Wilcoxon rank sum test to examine non-normally-distributed continuous variables, whereas categorical variables were analyzed using the Pearson chi-squared (^*χ*2^) test. Secondly, the incidence rates for ACM and TF were subsequently calculated based upon the time between the index date and the date of censor, death, or end of follow-up as the patient-months of follow-up. The log-rank test was also used to examine the therapeutic effects, with the survival curves then being charted based upon the Kaplan-Meier method. Both Cox proportional hazard regression and competing risks analysis were subsequently applied to examine the outcomes of ACM and TF between APD and CAPD patients, with the proportional hazard assumption being checked using the Schoenfeld residuals test. In the competing risks analysis, the hazard of death is analyzed regarding kidney transplant and TF as censoring and the analysis of TF hazard is performed with kidney transplant and death regarded as censoring. Loss of follow-up or recovery of residual renal function was extremely rare in this study because that the NHI in Taiwan is a compulsory program and the catastrophic illness of ESRD is required to be confirmed by two nephrologists. The hazard ratio (HR) estimates and 95% confidence intervals (CIs) were calculated according to time. The Cox proportional hazard model also measured the age-specific HRs (decades) for ACM and TF. Differences between groups were considered significant if the two-sided *p*-value was <0.05. All analyses were performed using SAS 9.3 software (SAS Institute Inc., Cary, NC, USA).

### Sensitivity analyses

In order to assess the robustness of our findings, we restricted the analysis to the sub-groups of patients so as to compare the therapeutic outcomes between those patients treated entirely with APD modality (pure APD patients, switching to CAPD modality for less than 60 days) and those treated entirely with CAPD modality (pure CAPD patients, switching to APD modality for less than 60 days). We also conducted a series of analyses defining APD/CAPD usage at intervals of 120 and 180 days after the index date to minimize any misclassification bias, with the APD or CAPD patients being regrouped according to whether or not they were in receipt of PD therapy via a cycler for at least 30 days after the intervals.

## Additional Information

**How to cite this article**: Tang, C.-H. *et al*. Do Automated Peritoneal Dialysis and Continuous Ambulatory Peritoneal Dialysis Have the Same Clinical Outcomes? A Ten-year Cohort Study in Taiwan. *Sci. Rep.* **6**, 29276; doi: 10.1038/srep29276 (2016).

## Supplementary Material

Supplementary Information

## Figures and Tables

**Table 1:** Baseline characteristics of the study patients before and after propensity-score matched cohorts.

Characteristics	Overall cohort					Matched cohort				
	APD (n = 2,346)		CAPD (n = 7,175)		*p*-value	APD (n = 2,287)		CAPD (n = 2,287)		*p*-value
Gender: men	1,224	(52.2)	3,183	(44.4)	<0.001	1,181	(51.6)	1,181	(51.6)	1.00
Age at cohort entry	53.6	[16.2]	53.5	[14.8]	0.81	53.8	[6.1]	53.9	[6.1]	0.89
<30	195	(8.3)	458	(6.4)		181	(7.9)	181	(7.9)	1.00
30–39	330	(14.1)	857	(11.9)		308	(13.5)	308	(13.5)	
40–49	446	(19.0)	1613	(22.5)		445	(19.5)	445	(19.5)	
50–59	533	(22.7)	1907	(26.6)		529	(22.9)	529	(22.9)	
60–69	426	(18.2)	1290	(18.0)		412	(18.0)	412	(18.0)	
≥70	416	(17.7)	1050	(14.6)		412	(18.0)	412	(18.0)	
Year at cohort entry					<0.001					1.00
2001	73	(3.1)	569	(7.9)		73	(3.2)	73	(3.2)	
2002	81	(3.5)	529	(7.4)		81	(3.5)	81	(3.5)	
2003	123	(5.2)	597	(8.3)		120	(5.3)	120	(5.3)	
2004	126	(5.4)	626	(8.7)		126	(5.5)	126	(5.5)	
2005	142	(6.1)	649	(9.1)		138	(6.0)	138	(6.0)	
2006	184	(7.8)	752	(10.5)		181	(7.9)	181	(7.9)	
2007	290	(12.4)	947	(13.2)		289	(12.6)	289	(12.6)	
2008	386	(16.5)	895	(12.5)		379	(16.6	379	(16.6	
2009	476	(20.3)	802	(11.2)		453	(19.8)	453	(19.8)	
2010	465	(19.8)	809	(11.3)		447	(19.6)	447	(19.6)	
Charlson comorbid index*	3.95	[1.88]	3.75	[1.91]	<0.001	3.94	[1.87]	3.85	[1.94]	0.08
No. of hospitalizations†	2.48	[1.90]	2.31	[1.91]	<0.001	2.47	[1.89]	2.39	[2.07]	0.20
Comorbidities, (ICD-9-CM codes)†										
Diabetes mellitus (250)	992	(42.3)	2658	(37.0)	<0.001	967	(42.3)	945	(41.3)	0.51
Hypertension (401–405)	2,096	(89.3)	6,104	(85.1)	<0.001	2,045	(89.4)	2,035	(89.0)	0.63
Cancer (140–208)	98	(4.2)	309	(4.3)	0.79	97	(4.2)	109	(4.8)	0.39
COPD (491–493, 495–496)	152	(6.5)	438	(6.1)	0.51	149	(6.5)	123	(5.4)	0.10
Gastric ulcer (531–534)	465	(19.8)	1287	(17.9)	0.04	451	(19.7)	402	(17.6)	0.06
Cirrhosis of liver (571)	151	(6.4)	520	(7.3)	0.18	145	(6.3)	181	(7.9)	0.04
Dementia (290)	15	(0.6)	65	(0.9)	0.22	13	(0.6)	19	(0.8)	0.29
Cerebrovascular disease (430–438)	237	(10.1)	636	(8.9)	0.07	230	(10.1	198	(8.7)	0.10
Peripheral vascular disease (440.2, 443)	31	(1.3)	107	(1.5)	0.55	31	(1.4)	31	(1.4)	1.00
Cardiac dysrhythmia (426, 427)	152	(6.5)	445	(6.2)	0.63	148	(6.5)	118	(5.2)	0.06
Ischemic heart disease (411, 413, 414)	477	(20.3)	1387	(19.3)	0.29	467	(20.4)	418	(18.3)	0.07
Myocardial infarction (410, 412)	66	(2.8)	187	(2.6)	0.59	73	(3.2)	54	(2.4)	0.09
Chronic heart failure (428)	408	(17.4)	1086	(15.1)	<0.01	394	(17.2)	373	(16.3)	0.41
Medications at cohort entry‡										
Anti-hypertension agents	2,254	(96.1)	6,784	(94.6)	<0.01	2,199	(96.2)	2,196	(96.0)	0.82
Anti-platelet agents	655	(27.9)	2128	(29.7)	0.11	640	(28.0)	633	(27.7)	0.82
Lipid-lowering agents	592	(25.2)	1531	(21.3)	<0.001	566	(24.8)	569	(24.9)	0.92
Oral hypoglycemic agents or insulins	961	(41.0)	2551	(35.6)	<0.001	937	(41.0)	921	(40.3)	0.63
Premium wage classes					<0.001					0.06
Class 1 ≤ USD 760	237	(10.1)	743	(10.4)		231	(10.1)	231	(10.1)	
Class 1 USD 761–1,210	243	(10.4)	645	(9.0)		233	(10.2)	207	(9.1)	
Class 1 USD 1,201–1,927	308	(13.1)	805	(11.2)		289	(12.6)	246	(10.8)	
Class 1 > USD 1,927	224	(9.6)	570	(7.9)		213	(9.3)	171	(7.5)	
Class 2-class 6	1334	(56.9)	4412	(61.5)		1321	(57.8)	1432	(62.6)	

Abbreviations: APD, automatic peritoneal dialysis; CAPD, continuous ambulatory peritoneal dialysis; COPD, chronic obstructive pulmonary disease; ICD-9-CM, International classification of diseases, 9^th^ revision, Clinical Modification. Data were number (%) or mean [standard deviation].

^*^Within 1 years before the index date.

^†^Within 2 years before the index date.

^‡^Within 3 months before the index date.

**Table 2:** Outcomes of the study patients after propensity-score matching.

APD vs. CAPD outcomes	APD				CAPD			
	No. of events	Exposure time, patient-month	Events/1000 patient-years	No. of events	Exposure time, patient-month	Events/1000 patient-years	RR	95% CI
2001–2010 (n = 2,287)								
All-cause mortality	514	69,151	89.2	470	73,487	76.7	1.16	1.03–1.32
Technique failure	354	69,151	61.4	334	73,487	54.5	1.13	0.97–1.31
Events of peritonitis	1,015	69,151	176.1	1,204	73,487	196.6	0.90	0.82–0.97
Kidney transplant	197	69,151	34.2	156	73,487	25.5	1.34	1.09–1.65
2001–2004 (n = 400)								
All-cause mortality	122	14,725	99.4	115	15390	89.7	1.11	0.86–1.43
Technique failure	72	14,725	58.7	102	15390	79.5	0.74	0.55–0.99
Events of peritonitis	237	14,725	193.1	307	15390	239.4	0.81	0.68–0.96
Kidney transplant	83	14,725	67.6	47	15,390	36.6	1.85	1.29–2.65
2005–2007 (n = 608)								
All-cause mortality	182	22,826	95.7	151	26,155	69.3	1.38	1.11–1.71
Technique failure	130	22,826	68.3	97	26,155	44.5	1.54	1.18–2.00
Events of peritonitis	365	22,826	191.9	389	26,155	178.5	1.08	0.93–1.24
Kidney transplant	65	22,826	34.2	53	26,155	24.3	1.41	0.98–2.03
2008–2010 (n = 1,279)								
All-cause mortality	210	31,600	79.7	204	31,942	76.6	1.04	0.86–1.26
Technique failure	152	31,600	57.7	135	31,942	50.7	1.14	0.90–1.44
Events of peritonitis	413	31,600	156.8	508	31,942	190.8	0.82	0.72–0.94
Kidney transplant	49	31,600	18.6	56	31,942	21.0	0.89	0.61–1.31

Abbreviations: APD, automatic peritoneal dialysis; CAPD, continuous ambulatory peritoneal dialysis; CI, confidence interval; HD, hemodialysis; RR, rate ratio.

**Table 3:** Cox proportional hazard analysis and competing risks analysis of APD and CAPD patients after propensity-score matching.

APD vs. CAPD outcomes	Cox proportional hazard analysis						Competing risks analysis					
	Univariate			Multivariate*			Univariate			Multivariate*,†		
	HR	95% CI	*p*-value	HR	95% CI	*p*-value	HR	95% CI	*p*-value	HR	95% CI	*p*-value
2001–2010 (n = 2,287)												
All-cause mortality	1.17	1.03–1.33	0.01	1.17	1.03–1.32	0.02	1.14	1.01–1.29	0.04	1.12	0.98–1.27	0.09
Technique failure	1.13	0.97–1.31	0.11	1.17	1.01–1.36	0.04	1.10	0.95–1.27	0.22	1.11	0.96–1.29	0.16
2001–2004 (n = 400)												
All-cause mortality	1.11	0.86–1.44	0.41	1.05	0.81–1.36	0.73	1.13	0.87–1.45	0.37	1.05	0.80–1.37	0.74
Technique failure	0.74	0.55–0.99	<0.05	0.70	0.52–0.95	0.02	0.73	0.54–0.98	0.04	0.71	0.52–0.97	0.03
2005–2007 (n = 608)												
All-cause mortality	1.40	1.13–1.74	<0.01	1.51	1.22–1.88	<0.001	1.29	1.04–1.60	0.02	1.37	1.09–1.72	<0.01
Technique failure	1.54	1.19–2.01	0.001	1.57	1.21–2.05	<0.001	1.43	1.10–1.86	<0.01	1.43	1.10–1.86	<0.01
2008–2010 (n = 1,279)												
All-cause mortality	1.04	0.86–1.27	0.66	0.98	0.81–1.19	0.83	1.03	0.85–1.25	0.73	0.98	0.80–1.19	0.83
Technique failure	1.14	0.90–1.43	0.28	1.21	0.96–1.53	0.11	1.13	0.90–1.43	0.29	1.15	0.92–1.45	0.22

Abbreviation: APD, automatic peritoneal dialysis; CAPD, continuous ambulatory peritoneal dialysis; CI, confidence interval; HR, risk ratio.

^*^The control variables included in the multivariate model were age, gender, diabetes mellitus, cirrhosis of liver, cerebrovascular disease, ischemic heart disease, chronic heart failure, events of peritonitis, icodextrin usage, and premium wage classes.

^†^Fine and Gray regression model.

**Table 4:** Cox proportional hazard analysis and competing risks analysis of pure APD and pure CAPD patients.

Pure APD vs. Pure CAPD outcomes	Cox proportional hazard analysis						Competing risks analysis					
	Univariate			Multivariate*			Univariate			Multivariate*,†		
	HR	95% CI	*p*-value	HR	95% CI	*p*-value	HR	95% CI	*p*-value	HR	95% CI	*p*-value
2001–2010, APD (n = 2,184) vs. CAPD (n = 2,244)												
All-cause mortality	1.20	1.06–1.36	<0.01	1.21	1.06–1.37	<0.01	1.16	1.02–1.32	0.02	1.15	1.01–1.31	0.04
Technique failure	1.16	0.99–1.35	0.05	1.21	1.04–1.41	0.02	1.13	0.97–1.31	0.13	1.15	0.98–1.33	0.08
2001–2004, APD (n = 369) vs. CAPD (n = 390)												
All-cause mortality	1.17	0.90–1.52	0.23	1.16	0.89–1.51	0.29	1.18	0.91–1.53	0.22	1.13	0.85–1.48	0.40
Technique failure	0.77	0.57–1.05	0.10	0.74	0.54–1.01	0.06	0.75	0.55–1.02	0.07	0.74	0.54–1.01	0.05
2005–2007, APD (n = 581) vs. CAPD (n = 595)												
All-cause mortality	1.43	1.15–1.78	0.001	1.54	1.24–1.93	0.001	1.31	1.06–1.63	0.01	1.39	1.11–1.75	<0.01
Technique failure	1.59	1.22–2.08	0.001	1.63	1.25–2.13	<0.001	1.47	1.12–1.91	<0.01	1.47	1.12–1.92	<0.01
2008–2010, APD (n = 1,234) vs. CAPD (n = 1,259)												
All-cause mortality	1.06	0.87–1.29	0.58	1.00	0.82–1.22	0.98	1.04	0.86–1.27	0.66	0.99	0.81–1.22	0.97
Technique failure	1.17	0.92–1.48	0.20	1.25	0.98–1.59	0.07	1.16	0.92–1.47	0.21	1.20	0.95–1.51	0.13

Abbreviation: APD, automatic peritoneal dialysis; CAPD, continuous ambulatory peritoneal dialysis; CI, confidence interval; HR, risk ratio.

^*^The control variables included in the multivariate model were age, gender, diabetes mellitus, cirrhosis of liver, cerebrovascular disease, ischemic heart disease, chronic heart failure, events of peritonitis, icodextrin usage, and premium wage classes.

^†^Fine and Gray regression model.
