# Supplementary material for: Do Automated Peritoneal Dialysis and Continuous Ambulatory Peritoneal Dialysis Have the Same Clinical Outcomes? A Ten-year Cohort Study in Taiwan
Source: Sci Rep. 2016 Jul 8;6:29276. doi: 10.1038/srep29276 (PMC4937348; doi:10.1038/srep29276)
Supplement: Supplementary Information [file srep29276-s1.pdf]

Supplementary Online Content

Do Automated Peritoneal Dialysis and Continuous Ambulatory Peritoneal Dialysis Have the Same Clinical Outcomes? A Ten-year Cohort Study in Taiwan

Tang CH, Chen TH, Fang TC, Huang SY, Huang KC, Wu YT, Wang CC, Sue YM

Table S1. The logistic regression model used to calculate propensity scores.

Table S2. Distribution of propensity scores among APD and CAPD patients in the full and matched cohorts.

Table S3. Annual peritoneal dialysis-related quantity of solution.

Table S4. Subgroup analysis of Cox proportional hazard regression for all-cause mortality and technique failure of APD and CAPD patients by cohort periods

Table S5. Hazard rates of all-cause mortality and technique failure in PD patients with delaying the index date to 120 days and regrouping the patients.

Table S6. Hazard rates of all-cause mortality and technique failure in PD patients with delaying the index date to 180 days and regrouping the patients.

Figure S1. Adjusted hazard ratios of APD to CAPD from the final Cox proportional hazard multivariate analysis for all-cause mortality and technique failure, by age groups.

This supplementary material has been provided by the authors to give readers additional information about their work.

**Table S1. The logistic regression model used to calculate propensity scores**					
Parameters	Estimate	Standard Error	Wald Chi-Square	OR (95% CI)	*p*-value
Intercept	-1.3869	0.1601	75.0184		<0.0001
Gender					
Male	0.2889	0.0489	34.891	1.335 (1.213-1.469)	<0.0001
Female	Ref				
Age at cohort entry, (y)					
<30	Ref				
30-39	-0.1735	0.1087	2.5455	0.841 (0.679-1.040)	0.1106
40-49	-0.5133	0.1031	24.8035	0.598 (0.489-0.732)	<0.0001
50-59	-0.5650	0.1028	30.1864	0.568 (0.465-0.695)	<0.0001
60-69	-0.3912	0.108	13.1101	0.676 (0.547-0.836)	0.0003
≥70	-0.1675	0.1122	2.2284	0.846 (0.679-1.054)	0.1355
Charlson comorbidity index					
0-2	Ref				
3-5	0.1739	0.0625	7.7548	1.190 (1.053-1.345)	0.0054
≥6	0.2592	0.0967	7.1811	1.296 (1.072-1.566)	0.0074
Comorbidities					
Diabetes mellitus	0.00285	0.0865	0.0011	1.003 (0.846-1.188)	0.9737
Hypertension	0.2827	0.0818	11.9547	1.327 (1.130-1.557)	0.0005
Dementia	-0.5253	0.2932	3.2103	0.591 (0.333-1.051)	0.0732
Cerebrovascular disease	-0.0029	0.0863	0.0012	0.997 (0.842-1.181)	0.9729
Peripheral vascular disease	-0.2388	0.2079	1.3194	0.788 (0.524-1.184)	0.2507
Cardiac dysrhythmia	-0.0061	0.1015	0.0037	0.994 (0.815-1.213)	0.9518
Ischemic heart disease	-0.0790	0.0679	1.3522	0.924 (0.809-1.056)	0.2449
Myocardial infarction	-0.0008	0.147	0	0.999 (0.749-1.333)	0.9957
Medications at cohort entry					
Anti-hypertension agents	0.0755	0.128	0.3477	1.078 (0.839-1.386)	0.5554
Anti-platelet agents	-0.1541	0.0556	7.6728	0.857 (0.769-0.956)	0.0056
Lipid-lowering agents	0.1625	0.0599	7.3553	1.176 (1.046-1.323)	0.0067
Oral hypoglycemic agents or insulins	0.1008	0.0768	1.7246	1.106 (0.952-1.286)	0.1891
Premiums wages classes					
Class 1 ≤ USD 760	Ref				
Class 1 USD 761-1210	0.1602	0.1072	2.2359	1.174 (0.951-1.448)	0.1348
Class 1 USD 1201-1927	0.1867	0.1018	3.3621	1.205 (0.987-1.471)	0.0667
Class 1 > USD 1927	0.2156	0.1107	3.7925	1.241 (0.999-1.541)	0.0515
Class 2-class 6	-0.0431	0.0822	0.2745	0.958 (0.815-1.125)	0.6004

CI, confidence interval; OR, odds ratio.

Table S2. Distribution of propensity scores among APD and CAPD patients in the full and matched cohorts

	Full cohort		Matched cohort	
	APD	CAPD	APD	CAPD
No. of observation	2,346	7,175	2,287	2,287
Mean	0.2604850	0.2417982	0.2527183	0.2486964
Standard deviation	0.0582170	0.0591351	0.0396343	0.0391438
Minimum	0.1214528	0.1044212	0.1488232	0.1450180
Lower quartile	0.2185823	0.1966938	0.2251867	0.2216093
Median	0.2566722	0.2378034	0.2541980	0.2521377
Upper quartile	0.2991247	0.2814910	0.2794834	0.2743920
Maximum	0.4651460	0.4394749	0.3711059	0.3845843

Table S3. Annual peritoneal dialysis-related quantity of solution

Quantity of solution (L)	APD (n=2,287)				CAPD (n=2,287)				*p*-value
	N	Mean	Median	SD	N	Mean	Median	SD	
Total quantity	2287	3530	3609	754	2287	2677	2751	670	<0.001*
1.5% Dextrose	2235	1782	1728	1041	2069	1428	1395	836	<0.001*
2.5% Dextrose	2211	1467	1474	964	2046	933	827	686	<0.001*
4.25% Dextrose	930	121	27	230	853	85	25	146	0.16†
Icodextrin	1333	434	502	265	1244	423	480	248	0.28*

Abbreviation: APD, automatic peritoneal dialysis; CAPD, continuous ambulatory peritoneal dialysis; SD, standard deviation.

*Student's t-test.

†Wilcoxon rank-sum test.

Table S4. Subgroup analysis of Cox proportional hazard regression for all-cause mortality and technique failure of APD and CAPD patients by cohort periods

	APD*			CAPD*		
Subgroups	HR	95% CI	*p*-value	HR	95% CI	*p*-value
All-cause mortality						
2001-2004, APD (n=400), CAPD (n=400)	As reference			As reference		
2005-2007, APD (n=608), CAPD (n=608)	0.89	0.69-1.15	0.33	0.69	0.53-0.90	<0.01
2008-2010, APD (n=1,279), CAPD (n=1,279)	0.74	0.56-0.98	0.03	0.72	0.55-0.93	0.01
Technique failure						
2001-2004, APD (n=400), CAPD (n=400)	As reference			As reference		
2005-2007, APD (n=608), CAPD (n=608)	1.21	0.88-1.65	0.25	0.56	0.41-0.76	<0.001
2008-2010, APD (n=1,279), CAPD (n=1,279)	0.85	0.61-1.19	0.85	0.63	0.47-0.85	<0.01

Abbreviation: APD, automatic peritoneal dialysis; CAPD, continuous ambulatory peritoneal dialysis; CI, confidence interval; HR, risk ratio.

*The control variables included in the multivariate model were age, gender, diabetes mellitus, cirrhosis of liver, cerebrovascular disease, ischemic heart disease, chronic heart failure, events of peritonitis, icodextrin usage, and premiums wages classes.

Table S5. Hazard rates of all-cause mortality and technique failure in PD patients with delaying the index date to 120 days and regrouping the patients

	Univariate			Multivariate*		
APD vs. CAPD outcomes	HR	95% CI	*p*-value	HR	95% CI	*p*-value
2001-2010, APD (n=2,165) vs. CAPD (n=2,377)						
All-cause mortality	1.13	0.99-1.28	0.07	1.14	1.00-1.15	0.05
Technique failure	1.13	0.97-1.31	0.13	1.17	1.00-1.36	0.05
2001-2004, APD (n=374) vs. CAPD (n=421)						
All-cause mortality	1.04	0.80-1.34	0.80	1.00	0.77-1.29	0.97
Technique failure	0.73	0.53-0.99	0.04	0.69	0.51-0.95	0.02
2005-2007, APD (n=562) vs. CAPD (n=644)						
All-cause mortality	1.33	1.07-1.65	0.01	1.48	1.19-1.84	<0.001
Technique failure	1.65	1.26-2.14	<0.001	1.68	1.29-2.19	0.001
2008-2010, APD (n=1,229) vs. CAPD (n=1,312)						
All-cause mortality	1.04	0.85-1.26	0.72	0.981	0.81-1.19	0.84
Technique failure	1.09	0.86-1.38	0.47	1.16	0.91-1.46	0.23

Abbreviation: APD, automatic peritoneal dialysis; CAPD, continuous ambulatory peritoneal dialysis; CI, confidence interval; HR, risk ratio.

*The control variables included in the multivariate model were age, gender, diabetes mellitus, cirrhosis of liver, cerebrovascular disease, ischemic heart disease, chronic heart failure, events of peritonitis, icodextrin usage, and premiums wages classes.

Table S6. Hazard rates of all-cause mortality and technique failure in PD patients with delaying the index date to 180 days and regrouping the patients

	Univariate			Multivariate*		
APD vs. CAPD outcomes	HR	95% CI	*p*-value	HR	95% CI	*p*-value
2001-2010, APD (n=2,017) vs. CAPD (n=2,387)						
All-cause mortality	1.19	1.04-1.35	<0.01	1.21	1.06-1.38	<0.01
Technique failure	1.10	0.94-1.28	0.26	1.14	0.97-1.34	0.11
2001-2004, APD (n=328) vs. CAPD (n=428)						
All-cause mortality	1.18	0.90-1.54	0.23	1.18	0.90-1.53	0.22
Technique failure	0.72	0.52-1.01	0.05	0.71	0.51-0.99	0.04
2005-2007, APD (n=531) vs. CAPD (n=639)						
All-cause mortality	1.17	1.05-1.31	<0.01	1.53	1.22-1.92	<0.001
Technique failure	1.16	1.01-1.33	0.03	1.40	1.07-1.84	0.02
2008-2010, APD (n=1,158) vs. CAPD (n=1,320)						
All-cause mortality	1.03	0.93-1.14	0.59	0.98	0.80-1.21	0.89
Technique failure	1.10	0.97-1.24	0.15	1.26	0.98-1.62	0.07

Abbreviation: APD, automatic peritoneal dialysis; CAPD, continuous ambulatory peritoneal dialysis; CI, confidence interval; HR, risk ratio.

*The control variables included in the multivariate model were age, gender, diabetes mellitus, cirrhosis of liver, cerebrovascular disease, ischemic heart disease, chronic heart failure, events of peritonitis, icodextrin usage, and premiums wages classes.

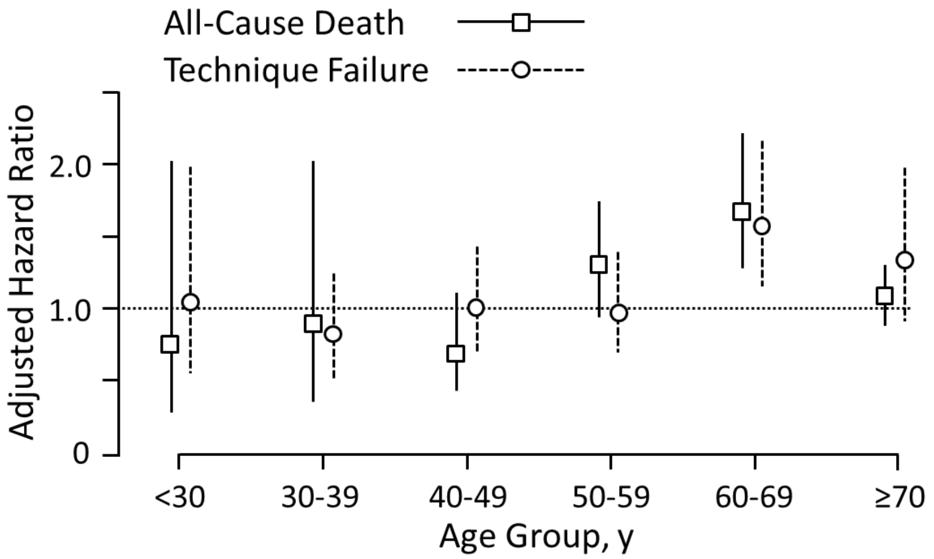

Figure S1. Adjusted hazard ratios of APD to CAPD from the final Cox proportional hazard multivariate analysis for all-cause mortality and technique failure, by age groups.
